# Time and Dose-Dependent Effects of *Labisia pumila* on Bone Oxidative Status of Postmenopausal Osteoporosis Rat Model

**DOI:** 10.3390/nu6083288

**Published:** 2014-08-21

**Authors:** Nadia Mohd Effendy, Ahmad Nazrun Shuid

**Affiliations:** Department of Pharmacology, Faculty of Medicine, Universiti Kebangsaan Malaysia, 50300 Kuala Lumpur, Malaysia; E-Mail: nadiaeffendy@yahoo.com

**Keywords:** osteoporosis, menopause, estrogen, antioxidant, *Labisia pumila*

## Abstract

Postmenopausal osteoporosis can be associated with oxidative stress and deterioration of antioxidant enzymes. It is mainly treated with estrogen replacement therapy (ERT). Although effective, ERT may cause adverse effects such as breast cancer and pulmonary embolism. *Labisia pumila var. alata* (LP), a herb used traditionally for women’s health was found to protect against estrogen-deficient osteoporosis. An extensive study was conducted in a postmenopausal osteoporosis rat model using several LP doses and duration of treatments to determine if anti-oxidative mechanisms were involved in its bone protective effects. Ninety-six female Sprague-Dawley rats were randomly divided into six groups; baseline group (BL), sham-operated (Sham), ovariectomised control (OVXC), ovariectomised (OVX) and given 64.5 μg/kg of Premarin (ERT), ovariectomised and given 20 mg/kg of LP (LP20) and ovariectomised and given 100 mg/kg of LP (LP100). The groups were further subdivided to receive their respective treatments via daily oral gavages for three, six or nine weeks of treatment periods. Following euthanization, the femora were dissected out for bone oxidative measurements which include superoxide dismutase (SOD), glutathione peroxidase (GPx) and malondialdehyde (MDA) levels. Results: The SOD levels of the sham-operated and all the treatment groups were significantly higher than the OVX groups at all treatment periods. The GPx level of ERT and LP100 groups at the 9th week of treatment were significantly higher than the baseline and OVX groups. MDA level of the OVX group was significantly higher than all the other groups at weeks 6 and 9. The LP20 and LP100 groups at the 9th week of treatment had significantly lower MDA levels than the ERT group. There were no significant differences between LP20 and LP100 for all parameters. Thus, LP supplementations at both doses, which showed the best results at 9 weeks, may reduce oxidative stress which in turn may prevent bone loss via its anti-oxidative property.

## 1. Introduction

Osteoporosis has been associated with many factors primarily aging and hormonal disturbances which mainly affects women. Osteoporosis is a silent, slowly progressive systemic skeletal disease that is, characterized by low bone mass and microarchitectural deterioration of bone tissue leading to increased bone fragility, and consequently increased risk of fractures [1]. As defined by the World Health Organization (WHO), osteoporosis occurs when bone mineral density (BMD) T score is more than 2.5 standard deviation units below the peak bone mass reference standard for young women [2]. This is a common condition affecting 30% of women and 12% of men at some point in their lifetimes [3]. Based on the main causes of osteoporosis which are aging and hormonal disturbances, postmenopausal women are greatly affected by this condition due to tremendous loss of estrogen after menopause.

Aging and hormonal disturbances in postmenopausal women can be related to oxidative stress. Previous studies have reported that oxidative stress is higher in postmenopausal women, indicating that antioxidant status may be related to estrogen deficit [4,5]. Oxidative stress is associated with osteoporosis and antioxidants may play an important role in suppressing the development of osteoporosis [6]. Due to the estrogen reduction following menopause, the body is subjected to a high level of free radicals and disruption of the oxidative stress defense system. This condition was shown to be associated with decreased bone mineral density and bone quality in numerous studies [7] Previous studies by Jagger *et al*. [8] and Almeida *et al*. [9] have confirmed that, estrogen deficiency generates a lowering of thiol antioxidant defences in rodent bone, resulting in accelerated bone loss. A study by Bellanti *et al*. [10], showed that estrogen deficiency caused a reduction in antioxidant enzymes such as superoxide dismutase (SOD) and glutathione peroxidase (GPx) which were counteracted by estrogen replacement.

Since estrogens are the major hormonal regulator of bone metabolism, it has been used widely for the treatment of postmenopausal osteoporosis. Due to the presence of estrogen receptors on osteoblast and osteoclast cells, estrogen acts directly via the activation of estrogen-receptor complex which will stimulate the osteoblast formation and induce osteoclast apoptosis [11]. Hence, estrogen replacement therapy (ERT) is effective in increasing sex hormone levels and improving bone mass as reported in many studies. Although effective, prolonged use of ERT may lead to many adverse effects such as breast cancer, cardiac infarction, stroke and pulmonary embolism [12]. To date, other forms of effective treatment of postmenopausal osteoporosis are the selective estrogen receptor modulators (SERMs) such as Raloxifene and biphosphonates such as alendronate and risedronate [13]. It was reported that Raloxifene is able to prevent bone loss as well as reduce risk of fractures in women with low bone mass [14]. Biphosphonates on the other hand have also been proven to be potent inhibitors of bone resorption. Prolonged use of all these anti-osteoporotic agents however may result in adverse effects such as thromboembolism, cataract, esophagitis and osteonecrosis of jaw bones [15].

Although conventional osteoporosis treatments are available, the use of natural remedies such as tocotrienol [16], soy [17] and blueberry [18] are on the rise. The effectiveness and adverse effects of natural remedies need to be investigated further. *Labisia pumila* (LP) or also known by the locals as Kacip Fatimah, Akar Fatimah, Pokok Pinggang and Belangkas Hutan [19] has been widely used by women for many generations. Its water extract is traditionally consumed by women to treat menstrual irregularities, promote uterine contraction and promote sexual health function [20]. LP was also reported to be effective against gonorrhoea, rheumatism and sickness in bones [21]. The mechanisms of LP are still unclear but it has been speculated that the health values of LP are contributed by its phytoestrogenic, anti-oxidative and anti-inflammatory properties [22]. A previous study by Nazrun *et al*., (2011) showed that supplementation of LP at the dose of 17.5 mg/kg was able to increase bone formation marker and reduce bone resorption marker in ovariectomized rats [23]. There is a paucity of reports in the literature on antioxidative mechanisms of LP although this herb is rich in anti-oxidant compounds. Since our main concern is oxidative stress-related osteoporosis, this study was conducted to determine the dose and time-dependent effects of LP supplementation on oxidative stress and anti-oxidative enzyme markers in the bone of ovariectomized rats.

## 2. Experimental Section

### 2.1. Animals and Treatment

The study was approved by the Animal Ethics Committee at the Universiti Kebangsaan Malaysia. Ninety-six female Sprague-Dawley rats aged 3–5 months weighing between 200 and 250 g were obtained from the Universiti Kebangsaan Malaysia Laboratory Animal Research Unit. The rats were housed in plastic cages at a temperature of 29 ± 3 °C under natural day/night cycle. They were fed with commercial food pellets (Gold Coin, Port Klang, Malaysia) and deionised water *ad libitum*. They were allowed to adjust to the new environment for a week before the study was started. They were then randomly divided into six main groups with six rats in the baseline group (BL) and eighteen rats in the rest of the groups which consisted of sham-operated (Sham), ovariectomized control (OVXC), ovariectomized and given estrogen (Premarin) at 64.5 μg/kg (ERT), ovariectomized and given *Labisia pumila* at 20 mg/kg (LP20) and ovariectomized and given *Labisia pumila* at 100 mg/kg (LP100). All the treatments were given daily via oral gavages. These groups were subdivided into three, six and nine weeks of treatment periods.

### 2.2. Labisia Pumila var. Alata (LP) and Estrogen (ERT)

The raw powdered form of LP was supplied by Delima Jelita Herbs (Alor Setar, Kedah, Malaysia). It was obtained from the *Labisia pumila var. alata* whole plant and was ground and freeze dried into powdered form. The dried powdered LP extract was sent to Forest Research Institute Malaysia (FRIM) for phytochemical screening to detect the phytochemical constituents. LP was dissolved in deionised water and given via oral gavage at doses of 20 mg/kg or 100 mg/kg rat weight daily at 9 am for 3, 6 or 9 weeks according to their assigned groups. Estrogen Premarin^®^ (Wyeth-Ayerst, Markham, Canada) tablet containing 0.625 mg of conjugated estrogen was crushed, dissolved in deionised water and given via oral gavage at the dose of 64.5 μg/kg rat weight daily at 9 am for 3, 6 or 9 weeks according to their assigned groups.

### 2.3. Bone Sampling

Rats in the BL group were euthanized before the start of the study while other rats were euthanized upon completion of their treatments. Femora were dissected and cleaned from all muscles and soft tissues. They were then wrapped in phosphate-buffered saline-soaked gauze and rewrapped with aluminum foil prior to storage in a −70 °C freezer until they were ready to be tested for superoxide dismutase (SOD), glutathione peroxidise (GPx) and malondialdehyde (MDA) level. SOD and GPx are the main enzymatic antioxidants produced by bone cells which play an important role in scavenging ROS [24] while MDA represents the end product of lipid peroxidation [25].

### 2.4. Measurement of Superoxide Dismutase (SOD) Enzyme

Measurement of SOD enzyme activity was performed using a Superoxide Dismutase Assay Kit from Cayman Chemical Company, Ann Arbor, MI, USA (catalogue No. 706002) [26]. The kit utilizes a tetrazolium salt for detection of superoxide radicals generated by xanthine oxidase and hypoxanthine. To prepare the tissue homogenate, firstly, femur was homogenized by perfusing with phosphate-buffered saline at pH 7.4 to remove any blood cells or clots. Then 0.25 g of bone was weighed and placed in a 10 mL tube containing 2 mL of 20 mM HEPES buffer (20 mM HEPES buffer pH 7.2, containing 1 mM EGTA, 210 mM Mannitol, and 70 mM sucrose per gram tissue). The mixture was then homogenized using tissue homogenizer Omni Bead Ruptor 24 (Omni International Incorporated, Kennesaw, GA, USA) prior to centrifugation at 1500× *g* for 5 min at 4 *°*C. The supernatant was collected to measure the absorbance spectrophotometrically at 540 nm using a Microplate Reader (VersaMax, Sunnyvale, CA, USA). The homogenized samples were diluted with sample buffer to bring the enzymatic activity to fall within the standard curve range. The standard curve of linearized rate (LR) of absorbance *versus* SOD activity was plotted, and the SOD activities in the samples were calculated. One unit of SOD is defined as the amount of enzyme needed to exhibit 50% dismutation of the superoxide radical.

### 2.5. Measurement of Glutathione Peroxidase (GPx)

Measurement of GPx enzyme activity was performed using Glutathione Peroxidase Assay Kit from Bioassay Systems, Hayward, CA, United States (catalogue No. EGPX-100). The kit indirectly measures nicotinamide adenine dinucleotide phosphate (NADPH) consumption in the enzyme coupled reaction. Oxidized glutathione (GSSG), produced upon reduction of hydroperoxide by GPx was recycled to its reduced state by glutathione reductase (GR) and NADPH. Prior to the assay procedure, about 10 mg femur was prepared for homogenization. The tissue was placed in a tube containing 0.2 mL phosphate-buffered saline prior to homogenization using an Omni Bead Ruptor 24 for 40 s. The homogenized tissue was centrifuged at 14,000× *g* for 10 min at 4 *°*C. The supernatant was finally collected to measure the absorbance spectrophotometrically at 340 nm using Microplate Reader. The samples were diluted with sample buffer to ensure that GPx activity was within the standard curve range. The optical density (OD) against standard concentrations was plotted to determine the slope of the standard curve. The measured decrease in OD at 340 nm is directly proportional to the enzyme activity of the bone sample. One unit of GPx is defined as the amount of enzyme that produces 1 μmol of GSSG per minute at 7.6 pH and room temperature [27].

### 2.6. Measurement of Malondialdehyde (MDA)

Measurement of MDA was performed using a TBARS Assay kit from Cayman Chemical Company, Ann Arbor, MI, USA. The measurement of Thiobarbituric Acid Reactive Substances (TBARS) is a well-established method for monitoring lipid peroxidation [28]. Prior to homogenization, about 25 mg of the femur was weighed and placed into tube containing 250 μL RIPA buffer with protease inhibitors. The mixture was then homogenized using Omni Bead Ruptor 24 for 40 s and centrifuged at 1600× *g* for 10 min at 4 °C. The supernatant was collected for analysis. The reaction of MDA and thiobarbituric acid (TBA) under high temperature, produced an MDA-TBA adduct which was measured colorimetrically at 540 nm. The standard curve of the MDA standards was plotted as a function of MDA concentration. The values of MDA of each sample were calculated from the standard curve. Lipids with higher unsaturation will yield higher MDA values [26].

### 2.7. Statistical Analysis

The data were analysed using Statistical Package for Social Sciences software (SPSS 19.0, Chicago, IL, USA). Firstly, the data were tested for normality using the Kolmogorov-Smirnov test (*n* ≤ 100). For normally distributed data, the statistical tests used were the analysis of variance (ANOVA), followed by Tukey’s HSD test. For data that were not normally distributed, Kruskal-Wallis and Mann-Whitney tests were used. All the results were expressed as mean ± standard error of the mean (SEM).

## 3. Results

### 3.1. Superoxide Dismutase (SOD)

**Figure 1:**
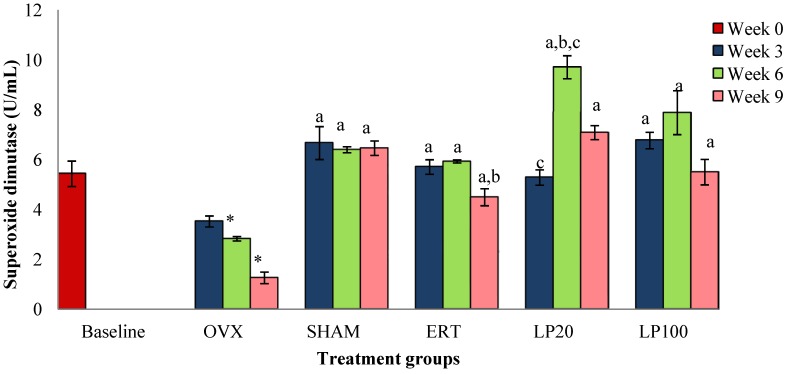
Mean superoxide dismutase concentration for all the groups after 3, 6 and 9 weeks of treatment. Data presented mean ± SEM (*p* < 0.05). SHAM: sham-operated, OVX: ovariectomized control, ERT: ovariectomized and estrogen supplementation, LP20: ovariectomized and supplemented with LP at the dose of 20mg/kg, LP100: ovariectomized and supplemented with LP at the dose of 100 mg/kg. * *p* < 0.05 *versus* Baseline; ^a^ *p* < 0.05 *versus* OVX at the corresponding weeks; ^b^ *p* < 0.05 *versus* SHAM at the corresponding weeks, ^c^ *p* < 0.05 *versus* ERT at week 6.

At the 3rd, 6th and 9th weeks all the groups showed significantly higher SOD level than the OVX group (*p* < 0.05). The SOD level of the OVX group was significantly lower than the BL group. The SOD level of the LP20 at 6th week was significantly higher than the SHAM and ERT groups, hence it exhibited the best result compared to the other groups (Figure 1).

### 3.2. Glutathione Peroxidase (GPx)

**Figure 2:**
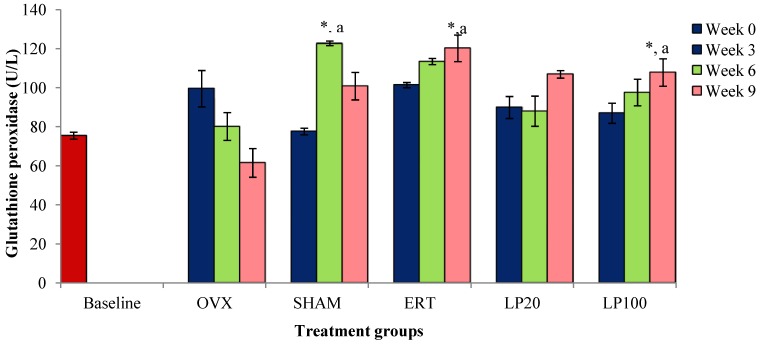
Mean glutathione peroxidase concentration for all the groups after 3, 6 and 9 weeks of treatment. Data presented mean ± SEM (*p* < 0.05). SHAM: sham-operated, OVX: ovariectomized control, ERT: ovariectomized and estrogen supplementation, LP20: ovariectomized and supplemented with LP at the dose of 20 mg/kg, LP100: ovariectomized and supplemented with LP at the dose of 100 mg/kg. * *p* < 0.05 *versus* Baseline; ^a^ *p* < 0.05 *versus* OVX at the corresponding weeks.

There was no significant result for all the groups at the 3rd week of treatment. At the 6th week, the SHAM group had a significantly higher GPx level compared to the BL and OVX groups. The ERT and LP100 groups at the 9th week showed higher GPx level than the BL and OVX groups (Figure 2).

### 3.3. Lipid Peroxidation (MDA)a

**Figure 3:**
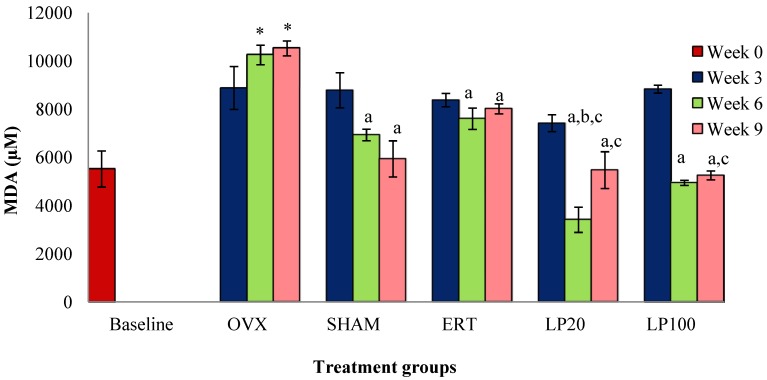
Mean melondialdehyde concentration for all the groups after 3, 6 and 9 weeks of treatment. Data presented mean ± SEM (*p* < 0.05). SHAM: sham-operated, OVX: ovariectomized control, ERT: ovariectomized and estrogen supplementation, LP20: ovariectomized and supplemented with LP at the dose of 20 mg/kg, LP100: ovariectomized and supplemented with LP at the dose of 100 mg/kg. * *p* < 0.05 *versus* Baseline; ^a^ *p* < 0.05 *versus* OVX at the corresponding weeks; ^b^ *p* < 0.05 *versus* SHAM at week 6; ^c^ *p* < 0.05 *versus* ERT at the corresponding weeks.

There was no significant result for all the groups at the 3rd week of treatment. The OVX group at the 6th and 9th weeks showed significantly higher MDA levels compared to the BL group. All the groups at the 6th and 9th weeks showed significantly lower MDA levels compared to the OVX group. The LP20 group at the 6th week had a significantly lower MDA level than the SHAM and ERT groups. LP20 and LP100 at the 9th week had significantly lower MDA levels compared to the ERT group (Figure 3).

## 4. Discussion

Estrogen exerts an antioxidant effect, which positively correlates with plasma antioxidant capacity and expression of antioxidant enzymes [29,30]. Simultaneously, estrogen correlates negatively with lipid peroxidation, a result of oxidative damage [31]. The antioxidant capacity of estrogen may be related to a direct free radical scavenging activity [32]. Nevertheless, the circulating level of estrogen is much lower than the necessary concentration of classical chemical antioxidants, indicating that its antioxidant effects is likely related to the upregulation of antioxidant enzymes [33]. Estrogen has profound effects on bone physiology, which keeps the bone turnover rate at balance. Estrogen reduction is the major cause of osteoporosis in postmenopausal women. Ovariectomized rats were used in this study because they exhibit progressive bone deterioration through a process that is similar to postmenopausal osteoporosis. Following ovariectomy, reduction in estrogen levels causes an increase in bone turnover, where bone resorption exceeds bone formation leading to bone loss [34].

Parallel with the antioxidant effect, reduction of estrogen following menopause may trigger oxidative stress, which consequently contributes to the mechanism of bone loss. It has been reported that oxidative stress and impairment of the antioxidant defense system are responsible for bone deterioration in postmenopausal women [35]. Reactive oxygen species (ROS) were shown to be responsible for the development of osteoporosis [36,37,38]. A previous study by Nazrun *et al*., (2011) has found that LP was able to increase the bone formation marker and reduce the bone resorption marker [23]. Other studies reported that there were improvements in histomorphometric parameters of ovariectomized rats supplemented with LP [39]. This has led to our interest in studying the antioxidative effect of LP on bone. To the best of our knowledge, this is the first report on an antioxidative mechanism of LP in preventing bone loss.

In our present study, two doses of LP (20 mg/kg and 100 mg/kg) were given at three different duration of treatments (3, 6 and 9 weeks) to evaluate the dose and time-dependent effects of LP on antioxidative parameters. LP extract has been shown to be safe with an LD_50_ of more than 5.0 g/kg [40]. According to other studies, LP extract was found to exhibit no-adverse-effect-level (NOAEL) at the dose of 50 mg/kg in sub-acute [41], 1000 mg/kg in sub-chronic [42] and 800 mg/kg in reproductive toxicity studies [40]. In human, the effective doses normally taken by women are around 500–1000 mg/kg daily. Therefore, the doses used in this study are considered to be safe.

Previously, the time-dependent effects of these two doses of LP (20 mg a/kg and 100 mg/kg) were investigated using *in vitro* micro-CT (micro computed tomography). It was shown that supplementation of LP 100 mg/kg for 9 weeks was the best treatment regimen to reverse the ovariectomy-induced bone changes compared to ERT and LP20 mg/kg. Bone volume fraction, connectivity density and trabecular number were increased and trabecular separation was decreased significantly in OVX rats treated with LP100 for 9 weeks. Three dimensional (3D) analysis also showed that the trabecular microarchitecture was improved significantly compared to other groups [43]. Following these positive results of LP on bone microarchitecture, our present study was performed to study one of the possible mechanisms of LP on bone.

At the 6th and 9th weeks of treatment, there were significant reductions in SOD levels of ovariectomised rats compared to baseline rats. These findings are consistent with a previous study by Muthusami *et al*. [44] which reported that OVX rats had a significant reduction in SOD enzyme. This indicates that deficiency in ovarian hormone correlates with failure to combat oxidative stress. Rats in all the groups at the 3rd, 6th and 9th weeks of treatment showed significantly higher SOD levels than the ovariectomised rats of the corresponding week. Both LP20 and LP100 groups showed significantly higher SOD than OVX. This is in line with a previous study in which LP was reported to have the ability to scavenge free radicals, which is contributed by the presence of high amount of phenolic compounds and flavonoids [45]. The free radical scavenging ability of LP supports our findings where SOD levels were higher in LP-supplemented rats.

Rats supplemented with ERT and LP100 for 9 weeks showed significantly higher GPx levels than rats in the OVX and BL groups. This reiterates the notion that in response to oxidative stress following ovariectomy, the levels of anti-oxidative enzymes were augmented by the antioxidative compounds present in LP [46]. Consistent with this, the MDA level of all the LP-supplemented groups were lowered. The ovariectomised rats on the other hand showed significantly higher MDA levels compared to other groups. Similar changes were seen in postmenopausal osteoporotic women where their glutathione reductase were significantly reduced while their MDA levels were significantly elevated compared to normal women [47]. SHAM group showed higher GPx and lower MDA at week 6 and 9. This is due to stimulation of antioxidant defenses to counteract oxidative dress during normal aging.

Following ovariectomy, reactive oxygen species (ROS) such as hydrogen peroxide (H_2_O_2_) were released in abundance together with cytokines and prostaglandins [48]. This may explain the oxidative stress findings in our study which were indicated by reductions in antioxidative enzymes and elevation in MDA levels in femur of OVX rats. Estrogen deficiency is known to interrupt the antioxidant defenses which in turn will upregulate oxidative stress. Oxidative stress may not only stimulate osteoclastic differentiation and function but it is also essential in osteoblast apoptosis [49,50]. Therefore, oxidative stress may lead to increased osteoclastic activity and decreased osteoblastic activity [51]. This will result in cell damage especially via lipid peroxidation, with MDA as the end product [52]. Lipid peroxidation may affect bone by stimulating osteoclasts differentiation. This is because osteoclasts express NADPH oxidase, which generates large quantities of ROS during bone resorption. Supplementation of both LP20 and LP100 were able to reduce MDA levels in femur of OVX rats. This is supported by previous study which revealed that LP has potential in scavenging free radicals [46]. Free radical scavenging ability of LP may indirectly inhibit the production of MDA.

Antioxidant enzymes play an important role in reducing lipid peroxidation by breaking down the oxidation chain and suppressing free radicals release [53]. Their levels would be reduced with the anti-oxidative activities as reported by the low SOD and GPx levels in the femur of OVX rats [44]. Another mechanism is via the activation of nuclear factor erythroid derived 2-related factor-2 (Nrf2) which plays an important role in cellular defense against oxidative stress. Nrf2 binds to the antioxidant response element (AFE), which subsequently induces anti-oxidative and phase-2 enzymes such as NADPH and glutathione s-transferase that are necessary for SOD and GPx biosynthesis. This activation of Nrf2 signalling pathway results in downregulation of oxidative stress [54,55]. ERT and LP-supplementation were found to maintain the antioxidant enzyme levels. This is in line with the fact that estrogen is able to offer antioxidant protection of lipoproteins and increase the expression of these enzymes in bone cells [56]. On the other hand, LP stimulated the antioxidant enzymes activity and decreased the MDA level via its phytoestrogenic and antioxidative properties [22]. Our findings may also be explained by free radical scavenging properties of LP as reported in the previous study [46].

Previous studies reported that the antioxidative properties of LP are contributed by the presence of flavonoids, ascorbic acid, beta-carotene, anthocyanin and phenolic compounds [57]. It was reported that among the constituents in LP, β-carotene showed the best correlation with the antioxidative activities, followed by flavonoids, ascorbic acid, anthocyanin and phenolic content [46]. β-carotene and flavonoids have been shown to be effective in scavenging free radicals by quenching singlet oxygen and consequently inhibiting peroxyl free radicals [58,59]. It was reported that flavonoids and phenolic compounds possess some features that resemble estrogens, allowing them to bind to the estrogen receptors (ERs) [60]. This binding will consequently regulate the receptors to stimulate osteoblasts activity [48]. The estrogen-like properties of these compounds may promote osteoblast differentiation and thus bone formation activities [49]. Chen *et al*., (2010) reported that phenolic acids were able to reduce osteoclastogenesis, hence reducing bone resorption activity as well as increasing bone mass [61]. There was also a report that rats supplemented with anthocyanin have higher osteoblast differentiation and osteoclast apoptosis [62]. In short, all these reports have strongly supported our study findings on the LP antioxidative effects on bone.

Oxidative stress may increase cytokine production by activating the transcription factors nuclear factor kappa B (NFκB) and activator protein-1 (AP-1) [63,64]. LP which contains flavonoid has been shown to inhibit production of nitric oxide (NO), via suppression of NFκB [65,66]. NO is an important regulator of bone metabolism [67,68]. It was reported that NO exerted biphasic effects on bone resorption, whereby high levels are associated with bone loss and vice versa [69]. High NO level contributes to the pathogenesis of osteoporosis by enhancing the ability of IL-1 and TNF to stimulate osteoclast activity [70]. LP may have prevented bone loss by suppressing the NO level via its antioxidative property. This is also supported by a study which has shown that the leaf and root extracts of *Labisia pumila var. alata* decreased NO production [71].

## 5. Conclusions

The present study has confirmed that LP has the potential to increase the anti-oxidative enzymes and reduce the oxidative stress in an estrogen-deficient postmenopausal rat model. Due to its comparable effects to ERT and good safety profile, LP has the potential to be used as an alternative treatment for prevention of postmenopausal osteoporosis. The mechanism may be contributed by an anti-oxidative property of LP; however, additional studies are needed to further clarify the mechanism of action of LP.

## References

[1] Christiansen C. (1993). Consensus development conference: Diagnosis, prophylaxis, and treatment of osteoporosis. Am. J. Med..

[2] Nelson H.D., Helfand M., Woolf S.H., Allan J.D. (2002). Screening for postmenopausal osteoporosis: A review of the evidence for the U.S. Preventive Services Task Force. Ann. Intern. Med..

[3] Laura A.G., Robert R.R. (2012). Pathophysiology of osteoporosis: New mechanistic insights. Endocrinol. Metab. Clin. N. Am..

[4] Signorelli S.S., Neri S., Sciacchitano S. (2006). Behaviour of some indicators of oxidative stress in postmenopausal and fertile women. Maturitas.

[5] Sanchez-Rodriguez M.A., Zacarias-Flores M., Arronte-Rosales A., Correa-Munoz E., Mendoza-Nunez V.M. (2011). Menopause as risk factor for oxidative stress. Menopause.

[6] Rao A.V., Rao L.G. (2007). Carotenoids and human health (Review). Pharmacol. Res..

[7] Zhang Y.B., Zhong Z.M., Hou G., Jiang H., Chen J.T. (2011). Involvement of oxidative stress in age-related bone loss. J. Surg. Res..

[8] Jagger C.J., Lean J.M., Davies J.T., Chambers T.J. (2005). Tumor necrosis factor mediates osteopenia caused by depletion of antioxidants. Endocrinology.

[9] Almeida M., O’Brien C.A. (2013). Basic biology of skeletal aging: Role of stress response pathways. J. Gerontol. Ser. A Biol. Sci. Med. Sci..

[10] Bellanti F., Matteo M., Rollo T., de Rosario F., Greco P., Vendemiale G., Serviddio G. (2013). Sex hormone modulate circulating antioxidant enzyme: Impact of estrogen therapy. Redox. Biol..

[11] Manolagas S.C., Kousteni S., Jilka R.L. (2002). Sex steroids and bone. Recent. Prog. Horm. Res..

[12] Wassertheil-Smoller S., Hendrix S.L., Limacher M., Heiss G., Kooperberg C., Baird A., Kotchen T., Curb J.D., Black H., Rossouw J.E. (2003). Effect of estrogen plus progestin on stroke in postmenopausal women: The Women’s Health Initiative: A randomized trial. JAMA.

[13] Reiner B., Bertha F., Emmo V.T., Christoph B. (2007). Biphosphonates in Medical Practice.

[14] Delmas P.D., Ensrud K.E., Adachi J.D., Harper K.D., Sarkar S., Gennari C., Reginster J. (2002). Efficacy of raloxifene on vertebral fracture risk reduction in postmenopausal women with osteoporosis: Four-year results from a randomized clinical trial. J. Clin. Endocr. Metab..

[15] Marcea M., Jia G., Theresa K., George B. (2012). Biphosphonates for Osteoporosis—Where do we go from here?. NEJM.

[16] Norliza M., Douglas A.L., Nazrun A.S., Norazlina M., Ima-Nirwana S. (2013). Tocotrienol supplementation in postmenopausal osteoporosis: Evidence from a laboratory study. Clinics.

[17] Arjmandi B.H., Lucas E.A., Khalil D.A., Devareddy L., Smith B.J., McDonalds J., Arquitt A.B., Payton M.E., Mason C. (2005). One year soy protein supplementation has positive effects on bone formation markers but not bone density in postmenopausal women. Nutr. J..

[18] Devareddy L., Hooshmad S., Collins J.K., Lucas E.A., Chai S.C., Arjmandi B.H. (2008). Blueberry prevents bone loss in ovariectomized rat model of postmenopausal osteoporosis. J. Nutr. Biochem..

[19] Jamia A.J., Houghton P.J., Milligan S.R., Ibrahim J. (1988). The Oestrogenic and Cytotoxic Effects of the Extracts of *Labisia pumila var. alata* and *Labisia pumila var. pumila in Vitro*. MJMS.

[20] Zakaria M., Mohd M.A. (1994). Traditional Malay MedicinalPlants.

[21] Jamal J.A., Houghton P.J., Milligan S.R. (1998). Testing of labisia pumila for oestrogenic activity using a recombinant yeast screen. J. Pharm. Pharmacol..

[22] Nadia M.E., Nazrun A.S., Norazlina M., Isa N.M., Norliza M., Ima-Nirwana S. (2012). The anti-inflammatory, phytoestrogenic and antioxidative role of *Labisia pumila* in prevention of postmenopausal osteoporosis. Adv. Pharmacol. Sci..

[23] Nazrun A.S., Lee P.L., Norliza M., Norazlina M., Ima-Nirwana S. (2011). The effects of *Labisia pumila var. alata* on bone markers and bone calcium in a rat model of post-menopausal osteoporosis. J. Ethnopharmacol..

[24] Mlakar S.J., Osredkar J., Prezeli J., Marc J. (2012). Antioxidant enzymes GSR, SOD1, SOD2 and Cat gene variants and bone mineral density values in postmenopausal women: A genetic association analysis. Menopause.

[25] Yagi K. (1998). Simple assay for the level of total lipid peroxides in serum or plasma. Method. Mol. Biol..

[26] Cayman Chemical Company. http://www.caymanchem.com/.

[27] BioAssay Systems EnzyChrom™ Glutathione Peroxidase Assay Kit. http://www.bioassaysys.com/.

[28] Armstrong D., Browne R. (1994). The analysis of free radicals, lipid peroxides, antioxidant enzymes and compounds to oxidative stress as applied to the clinical chemistry laboratory. Adv. Exp. Med. Biol..

[29] Bednarek-Tupikowska G., Bohdanowicz-Pawlak B., Bidzinska A., Melewicz J., Antonowicz-Juchniewicz R., Andrzejak R. (2001). Serum lipid peroxide levels and erythrocyte glutathione peroxidase and superoxide dismutase activity in premenopausal and postmenopausal women. Gynecol. Endocrinol..

[30] Serviddio G., Loverro G., Vicino M., Prigigallo F., Grattagliano I., Altomare E., Vendermiale G. (2002). Modulation of endometrial redox balance during the menstrual cycle: Relation with sex hormones. J. Clin. Endocrinol. Metab..

[31] Chang S.P., Yang W.S., Lee S.K., Min W.K., Park J.S., Kim S.B. (2002). Effects of hormonal replacement therapy on oxidative stress and total antioxidant capacity in postmenopausal hemodialysis patients. Ren Fail..

[32] Ruiz-Larrea M.B., Leal A.M., Martin C., Martinez R., Lacort M. (1997). Antioxidant action of estrogens in rat hepatoctes. Rev. Esp Fisiol..

[33] Borras C., Gambini J., Gomez-Cabrera M.C., Sastre J., Pallardó F.V., Mann G.E., Viña J. (2005). 17 beta-oestradiol up-regulates longevity-related, antioxidant enzyme expression via the ERK1 and ERK2^[MAPK]^/NFκB cascade. Aging Cell.

[34] Pavlos P.L., Theodoros T.X., Sofia E.T., George P.L., Ismene A.D. (2008). The laboratory rat as an animal model for osteoporosis research. Comp. Med..

[35] Lean J.M., Jagger C.J., Kirstein B., Fuller K., Chambers T.J. (2005). Hydrogen peroxide is essential for estrogen-deficiency bone loss and osteoclast formation. Endocrinology.

[36] Smietana M.J., Arruda E.M., Faulkner J.A., Brooks S.V., Larkin L.M. (2010). Reactive oxygen species on bone mineral density and mechanics in Cu, Zn superokxide dismutase (SOD1) knockout mice. Biochem. Biophys. Res. Commun..

[37] Afonso V., Champy R., Mitrovic D., Collin P., Lomri A. (2007). Reactive oxygen species and superoxide dismutases: Role in joint diseases. Joint Bone Spine.

[38] Ozgocmen S., Kaya H., Fadillioglu E., Aydogan R., Yilmaz Z. (2007). Role of antioxidant systems, lipid peroxidation, and nitric oxide in postmenopausal osteoporosis. Mol. Cell Biochem..

[39] Fathilah S.N., Nazrun A.S., Norazlina M., Norliza M., Ima-Nirwana S. (2012). *Labisia pumila* protects the bone of estrogen-deficient rat model: A histomorphometric study. J. Ethnopharmacol..

[40] Wan Ezumi M.F., Siti Amrah S., Suhaimi A.W.M., Mohsin S.S.J. (2007). Evaluation of the female reproductive toxicity of aqueous extract of *Labisia pumila var. alata* in rats. Indian J. Pharmacol..

[41] Singh G.D., Ganjoo M., Youssouf M.S., Koul A., Sharma R., Singh S., Sangwan P.L., Koul S., Ahamad D.B., Johri R.K. (2009). Sub-acute toxicity evaluation of an aqueous extract of *Labisia pumila*, a Malaysian herb. Food Chem. Toxicol..

[42] Taneja S.C. (2004). Sub-Chronic (90 days) Oral Toxicity Studies of Aqueous Extract of Labisia pumila in Wistar Rats (250,500 & 1000 mg/kg b. wt. only).

[43] Nadia M.E., Fadhli M.K., Ima Nirwana S., Nazrun A.S. (2014). The fffects of *Labisia pumila* on postmenopausal osteoporotic rat model: Dose and time-dependent micro-CT analysis. J. X-Ray Sci. Technol..

[44] Muthusami S., Ramachandran I., Muthusamy B., Vasudevan G., Prabhu V., Subramaniam V., Jagadeesan A., Narasimhan S. (2005). Ovariectomy induces oxidative stress and impairs bone antioxidant system in adult rats. Clin. Chim. Acta.

[45] Fazliana M. (2010). Studies on Labisia pumila var. alata Extract with Phytoestrogenic Effects: Impact on Biological Activities and Gene Expression.

[46] Norhaiza M., Maziah M., Hakiman M. (2009). Antioxidative properties of leaf extracts of a popular Malaysian herb, *Labisia pumila*. J. Med. Plants Res..

[47] Omer F.S., Yasemin T., Engin T., Mukadder S. (2009). Antioxidant status in patients with osteoporosis: A controlled study. Joint Bone Spine.

[48] Zhang L., Fujii S., Kosaka H. (2007). Effect of oestrogen on reactive oxygen species production in the aortas of ovariectomized Dahl salt-sensitive rats. J. Hypertens..

[49] Steinbeck M.J., Appel W.H., Verhoeven A.J., Karnovsky M.J. (1994). NADPH-oxidase expression and *in situ* production of superoxideby osteoclasts actively resorbing bone. J. Cell Biol..

[50] Sontakke A.N., Tare R.S. (2002). A duality in the roles of reactive oxygen species with respect to bone metabolism. Clin. Chim. Acta.

[51] Atlindag O., Erel O., Soran N., Celik H., Selek S. (2008). Total oxidative/anti-oxidative status and relation to bone mineral density in osteoporosis. Rheum Int..

[52] Niedernhofer L.J., Daniels J.S., Rouzer C.A., Greene R.E., Marnett L.J. (2003). Malondialdehyde, a product of lipid peroxidation, is mutagenic in human cells. J. Biol. Chem..

[53] Niki E., Yoshida Y., Saito Y., Noguchi N. (2005). Lipid peroxidation: Mechanisms, inhibition and biological effects. Biochem. Biophys. Res. Commun..

[54] Zhu H., Zhang L., Itoh K. (2006). Nrf2 controls bone marrow stromal cell susceptibility to oxidative and electrophilic stress. Free Radic Biol. Med..

[55] Susanne P., Sonja K., Mahmoud K. (2012). Nrf2/ARE signaling pathway: Key mediator in oxidative stress and potential therapeutic target in ALS. Neurol. Res. Int..

[56] Duke P., Sandy S. (2003). Thiol antioxidants reduce bone loss in estrogen-deficient female mice. Life Enhancement.

[57] Lee S.C., Norliza A.L., Sze Y.L., Chew T.L., Mohamad R.S., Ramlan A.A. (2011). Flavonoids and phenolic acids from *Labisia pumila* (Kacip Fatimah). Food Chem..

[58] Sies H., Stahl W. (1995). Vitamins E and C, β-carotene, and other carotenoids as antioxidants. Am. J. Clin. Nutr..

[59] Duthie G.G., Gardner P.T., Kyle J.A.M. (2003). Plant polyphenols: Are they the new magic bullet?. Proc. Nutr. Soc..

[60] Anderson J.J.B., Anthony M., Messina M., Garner S.C. (1999). Effects of phytoestrogens on tissues. Nutr. Res. Rev..

[61] Chen J.R., Lazarenko O.P., Wu X., Kang J., Blackburn M.L., Shankar K., Badger T.M., Ronis M.J. (2010). Dietary-induced serum phenolic acids promote bone growth via p38 MAPK/beta-catenin canonical wnt signalling. J. Bone Miner Res..

[62] Moriwaki S., Suzuki K., Muramatsu M., Nomura A., Inoue F., Into T., Yoshiko Y., Niida S. (2014). Delphinidin, one of the major anthocyanidins, prevents bone loss through the inhibition of excessive osteoclastogenesis in osteoporosis model. PLoS One.

[63] Kim S.J., Angel P., Lafyatis R., Hattori K., Kim K.Y., Sporn M.B., Karin M., Roberts A.B. (1990). Autoinduction of transforming growth factor beta 1 is mediated by the AP-1 complex. Mol. Cell Biol..

[64] Meyer M., Schreck R., Baeuerle P.A. (1993). H_2_O_2_ and antioxidants have opposite effects on activation of NF-kappa B and AP-1 in intact cells: AP-1 as secondary antioxidant-responsive factor. EMBO J..

[65] Lin Y.L., Lin J.K. (1997). Epigallocatechin-3-gallate blocks the induction of nitric oxide synthase by down-regulating lipopolysaccharide-induced activity of transcription factor nuclear factor-kappaB. Mol. Pharmacol..

[66] Gonzalez-Gallego J., Sanchez-Campoz S., Tunon M.J. (2007). Anti-inflammatory properties of dietary flavonoids. Nutr. Hosp..

[67] Van’t Hof R.J., Armour K.J., Smith L.M., Armour K.E., Wei X.Q., Liew F.Y., Ralston S.H. (2000). Requirement of the inducible nitric oxide synthase pathway for IL-1-induced osteoclastic bone resorption. Proce. Natl. Acad. Sci. USA..

[68] Hao Y.J., Tang Y., Chen F.B., Pei F.X. (2005). Different doses of nitric oxide donor prevent osteoporosis in ovariectomized rats. Clin. Orthop. Relat. Res..

[69] Armour K.E., Van’T Hof R.J., Grabowski P.S., Reid D.M., Ralston S.H. (1999). Evidence for a pathogenic role of nitric oxide in inflammation-induced osteoporosis. J. Bone Miner Res..

[70] Van’t Hof R.J., Ralston S.H. (2001). Nitric oxide and bone. Immunology.

[71] Ehsan K., Hawa Z.E.J., Syahida A. (2013). Antifungal, anti-inflammatory and cytotoxicity activities of three varieties of labisia pumila benth: From microwave obtained extracts. BMC Complement Altern Med..

